# Supplementary material for: Preventing suicide by restricting access to Highly Hazardous Pesticides (HHPs): A systematic review of international evidence since 2017
Source: PLOS Glob Public Health. 2025 Feb 3;5(2):e0003785. doi: 10.1371/journal.pgph.0003785 (PMC11790168; doi:10.1371/journal.pgph.0003785)
Supplement: S1 File — (DOCX) [file pgph.0003785.s002.docx]

**Supplementary materials**

**Additional methods**

**Box S1.** Search strategy terms and structure.

1 (Pesticide? or insecticide? or rodenticide? or fungicide? or herbicide? or paraquat or organophosphate? or organophosphorus or agricultural or agrochemical?).af.

2 (suicid?? or (self and harm) or parasuicide or (Self-Injurious and Behavior) or ((self? or deliberate) and poison???)).af.

3 1 and 2

4 limit 3 to yr="2017 -Current"

5 remove duplicates from 4

**Risk of bias assessment tool.** EPOC - Effective Practice and Organisation of Care (EPOC). Suggested risk of bias criteria for EPOC reviews. EPOC Resources for review authors. Oslo: Norwegian Knowledge Centre for the Health Services; 2015. Available at: <http://epoc.cochrane.org/epoc-specific-resources-review-authors> (1).

| **Domain** | **Risk of bias** | **Comment** |
| --- | --- | --- |
| **Was the intervention independent of other changes?**  Score “Low risk” if there are compelling arguments that the intervention occurred independently of other changes over time and the outcome was not influenced by other confounding variables/historic events during study period (esp. war, recession, alcohol bans, suicide coding changes). If Events/variables identified, note what they are. Score “High risk” if reported that intervention was not independent of other changes in time. | High / Low/unclear |  |
| **Was the shape of the intervention effect pre-specified?**  Score “Low risk” if point of analysis is the point of intervention OR a rational explanation for the shape of intervention effect was given by the author(s). Where appropriate, this should include an explanation if the point of analysis is NOT the point of intervention. Score “High risk” if it is clear that the condition above is not met. | High / Low/unclear |  |
| **Was the intervention unlikely to affect data collection?**  Score “Low risk” if reported that intervention itself was unlikely to affect data collection (for example, sources and methods of data collection were the same before and after the intervention); Score “High risk” if the intervention itself was likely to affect data collection (for example, any change in source or method of data collection reported). | High / Low/unclear |  |
| **Was knowledge of the allocated interventions adequately prevented during the study?**  Score “Low risk” if the authors state explicitly that the primary outcome variables were assessed blindly, or the outcomes are objective, e.g. length of hospital stay. Primary outcomes are those variables that correspond to the primary hypothesis or question as defined by the authors. Score “High risk” if the outcomes were not assessed blindly. Score “Unclear risk” if not specified in the paper. | High / Low/unclear |  |
| **Were incomplete outcome data adequately addressed?**  Score “Low risk” if missing outcome measures were unlikely to bias the results (e.g. the proportion of missing data was similar in the pre- and post-intervention periods or the proportion of missing data was less than the effect size i.e. unlikely to overturn the study result). Score “High risk” if missing outcome data was likely to bias the results. Score “Unclear risk” if not specified in the paper (Do not assume 100% follow up unless stated explicitly). | High / Low/unclear |  |
| **Was the study free from selective outcome reporting?**  Score “Low risk” if there is no evidence that outcomes were selectively reported (e.g. all relevant outcomes in the methods section are reported in the results section). Score “High risk” if some important outcomes are subsequently omitted from the results. Score “Unclear risk” if not specified in the paper. | High / Low/unclear |  |
| **Was the study free from other risks of bias?** Score “Low risk” if there is no evidence of other risk of biases. e.g. should consider if seasonality is an issue (i.e. if January to June comprises the preintervention period and July to December the post, could the “seasons’ have caused a spurious effect). | High / Low/unclear |  |
| **Was the same source of data used for pre- and post-ban rates of pesticide suicide and overall suicide and the legal definition of suicide the same over the study period?** If yes score Low risk; if No score high risk. Score “Unclear risk” if not specified in the paper. | High / Low/unclear |  |
| **Was any information given about whether the regulations were effective in reducing access to key high-toxicity pesticides (e.g. Score low risk if evidence of effect (e.g. reduced sales volume)?** Score “high risk” if evidence that the regulation was ineffective (e.g. because other sources of the pesticide were accessed by cross-border trade in small countries; use of the pesticide in self-harm was relatively unchanged after the restriction. Score “Unclear risk” if no evidence about effectiveness of the bans was specified in the paper. | High / Low/unclear |  |
| **Did the study ignore secular (trend) changes and perform a simple t-test of the pre versus post intervention periods without further justification?** If no evidence preceding trends were taken into account score “high risk”. If preceding trends taken into account score low risk. If the assessment was purely graphical score as unclear, there was no formal statistical comparison score as “no formal comparison” | High / Low / no formal comparison |  |

**References**

1. Higgins JPT, Thomas J, Chandler J, Cumpston M, Li T, Page MJ, Welch VA (editors). Cochrane Handbook for Systematic Reviews of Interventions version 6.4 (updated August 2023). Cochrane, 2023. Available from www.training.cochrane.org/handbook (accessed Arpil 30, 2024).
