# Supplementary material for: Preventing suicide by restricting access to Highly Hazardous Pesticides (HHPs): A systematic review of international evidence since 2017
Source: PLOS Glob Public Health. 2025 Feb 3;5(2):e0003785. doi: 10.1371/journal.pgph.0003785 (PMC11790168; doi:10.1371/journal.pgph.0003785)
Supplement: S1 Table — (DOCX) [file pgph.0003785.s003.docx]

**S1 Table.** Studies identified in the systematic search, with reasons for exclusion.

| **Authors** | **Title** | **Decision** | **Main reason  for exclusion** | **Comments** |
| --- | --- | --- | --- | --- |
| **Possible inclusions** |  |  |  |  |
| Ayra, V. et al. | Changes in method specific suicide following a national pesticide ban in India (2011-2014) | Include |  |  |
| Cha, E. S. et al. | Trends in pesticide suicide in South Korea, 1983-2014 | Include |  |  |
| Chang, S. S. et al. | The early impact of paraquat ban on suicide in Taiwan | Include |  |  |
| Chowdhury, F. B. et al. | Bans of WHO Class I pesticides in Bangladesh-suicide prevention without hampering agricultural output | Exclude | Other (specify on comments) | Included in previous SR |
| Eddleston, M. et al. | Pesticide use, agricultural outputs, and pesticide poisoning deaths in Japan | Include |  |  |
| Gao, Y. et al. | Epidemiological analysis of pesticide poisoning in Tianjin from 2009 to 2018 | Exclude | Wrong outcome |  |
| Indira, M. et al. | Effect of pesticide ban on suicide trend - a 20-year study from a tertiary care center in Central Kerala from 2001 to 2020 | Exclude | Selected population |  |
| Kim, H. et al. | Implementation and outcomes of suicide-prevention strategies by restricting access to lethal suicide methods in Korea | Exclude | Not original research |  |
| Kim, H. et al. | Effect of prohibiting the use of Paraquat on pesticide-associated mortality | Include |  |  |
| Knipe, D. et al. | Suicide prevention through means restriction: impact of the 2008-2011 pesticide restrictions on suicide in Sri Lanka | Exclude | Other (specify on comments) | Included in previous SR |
| Knipe, D. et al. | Preventing deaths from pesticide self-poisoning-learning from Sri Lanka's success | Exclude | Wrong outcome |  |
| Leong, Y-H. et al. | Paraquat poisoning calls to the Malaysia National Poison Centre following its ban and subsequent restriction of the herbicide from 2004 to 2015 | Exclude | Selected population |  |
| Perananthan, V. et al. | The clinical toxicity of imidacloprid self-poisoning following the introduction of newer formulations | Exclude | Wrong outcome |  |
| Sharma, D. et al. | Regulation of highly hazardous pesticides for pesticide suicide prevention in Nepal | Exclude | No intervention |  |
| Utyasheva, L. et al. | Suicide by pesticide ingestion in Nepal and impact of pesticide regulation | Exclude | Not original research |  |
| Wu, K.C. et al. | Regulatory control of highly hazardous pesticides to prevent self-poisoning | Exclude | Not original research |  |
|  |  |  |  |  |
| **Conflicts** |  |  |  |  |
| Armstrong, G. et al. | Suicide in India: a complex public health tragedy in need of a plan | Exclude | Not original research |  |
| Arya, V. et al. | Suicide by hanging is a priority for suicide prevention: method specific suicide in India (2001-2014) | Exclude | No comparison group |  |
| Bando, D et a. | Geographical clusters and social risk factors for suicide in the city of Sao Paulo, 2006-2015: An ecologic study | Exclude | Wrong outcome |  |
| Bonvoisin, T. et al. | Suicide by pesticide poisoning in India: a review of pesticide regulations and their impact on suicide trends | Include |  |  |
| Dabholkar, S. et al. | Suicides by pesticide ingestion in Pakistan and the impact of pesticide regulation | Exclude | Not original research |  |
| Dandona, R. et al. | Pesticide surveillance and deaths by suicide | Exclude | Not original research |  |
| Eddleston, M. et al. | Response to Bayer regarding pesticide suicides | Exclude | Not original research |  |
| Hulse, E. | Self-harm and poisoning data in rural Ugandan hospitals | Exclude | Not original research |  |
| Jaacks, L. M. et al. | Impact of large-scale, government legislated and funded organic farming training on pesticide use in Andhra Pradesh, India: a cross-sectional study | Exclude | Wrong outcome |  |
| Kim, Y. et al. | Suicide Overall and Suicide by Pesticide Rates among South Korean Workers: A 15-Year Population-Based Study | Exclude | Selected population |  |
| Lee, Y. Y. et al. | The cost-effectiveness of banning highly hazardous pesticides to prevent suicides due to pesticide self-ingestion across 14 countries: an economic modelling study | Exclude | Wrong outcome |  |
| Miller, G. | Pathways to prevention | Exclude | Not original research |  |
| Paik, J. W. | Suicide prevention action plan and post suicide attempt case management in South Korea | Exclude | Not original research |  |
| Pirkis, J. et al. | Suicide prevention in the Western Pacific region | Exclude | Not original research |  |
| Pirkis, J. et al. | Evaluating suicide prevention activities | Exclude | Not original research |  |
| Sathyanarayana Rao, T. S. et al. | Prevention of farmer suicides: Greater need for state role than for a mental health professional's role | Exclude | Not original research |  |
| Schess, J. et al. | The cost-effectiveness of a suicide prevention intervention restricting access to pesticides in india | Exclude | Wrong outcome |  |
| Silverman, M. M. et al. | Suicide Prevention in the Americas | Exclude | Not original research |  |
| Weerasinghe, M. et al. | Vendor-based restrictions on pesticide sales to prevent pesticide self-poisoning - a pilot study | Exclude | Wrong outcome |  |
| Weerasinghe, Met al. | Emerging pesticides responsible for suicide in rural Sri Lanka following the 2008-2014 pesticide bans | Exclude | No intervention |  |
| Widger, T. | Suicides, poisons and the materially possible: The positive ambivalence of means restriction and critical-critical global health | Exclude | Not original research |  |
| Wijerathna, T. M. et al. | International travel grant epidemiology following 2-methyl-4-chlorophenoxyacetic acid (MCPA) poisoning | Exclude | Selected population |  |
|  |  |  |  |  |
| **Maybe** |  |  |  |  |
| Abu Bakar, N. S. et al. | Trends of completed suicide rates among Malaysian elderly between 1995 and 2020 | Exclude | Selected population |  |
| Abubaker, Z. J. et al. | A retrospective analysis on poison related mortalities in a tertiary care centre in pakistan | Exclude | Selected population |  |
| Adams, R. D. et al. | 352. Enhanced monitoring of glyphosate exposures by TOXBASE: the National Poisons Information Service (NPIS) pesticide surveillance project 2004-2018 | Exclude | No intervention |  |
| Akhgari, M. et al. | Analysis of intoxication deaths: Causes and manners of death | Exclude | No intervention |  |
| Al Houri, H. N. et al. | Epidemiology of poisoning in Syria (1999 through 2020) | Exclude | Wrong intervention (e.g. lockable boxes, village stores, manufacturer initiaves) |  |
| Alahakoon, C. et al. | Differences between organophosphates in respiratory failure and lethality with poisoning post the 2011 bans in Sri Lanka | Exclude | Selected population |  |
| Amar, W. B. et al. | Fatal poisoning in south Tunisia: A 12 years study | Exclude | No intervention |  |
| Arafat, S. | Females are dying more than males by suicide in Bangladesh | Exclude | Not original research |  |
| Bachmann, S. | Perspectives of epidemiology | Exclude | Not original research |  |
| Beautrais, A. L. | Farm suicides in New Zealand, 2007-2015: A review of coroners' records | Exclude | No intervention |  |
| Behere, P. B. et al. | Suicide and self-harms in rural setting: With special reference to farmers' suicide | Exclude | Not original research |  |
| Behere, P. B. et al. | Suicidal Ideation and Pesticide Exposure in Rural Communities of Central India | Exclude | Wrong outcome |  |
| Betz, M. E. et al. | Firearms, pesticides, and suicide: A look back for a way forward | Exclude | Not original research |  |
| Bhattrai, S. et al. | Health Impact Associated with Pesticides Use among Vegetables Farmers in Nepal: A Secondary Analysis | Exclude | Selected population |  |
| Biradar, G. et al. | Profile of rodenticide poisoning at vijayanagar institute of medical sciences (Vims), ballari district, karnataka, india: Retrospective analysis of cases from 2016 to 2020 | Exclude | No intervention |  |
| Bochner, R. et al. | [Analysis of deaths by intoxication that occurred in Brazil from 2010 to 2015 based on the Mortality Information System (SIM)] | Exclude | No intervention |  |
| Borges-Santos, D. et al. | Suicide by hanging in Brazil: Challenges to mitigating its escalation | Exclude | Not original research |  |
| Buckley, N. A. et al. | Case fatality of agricultural pesticides after self-poisoning in Sri Lanka: a prospective cohort study | Exclude | Selected population |  |
| Buendia, J. A. et al. | Burden of paraquat poisoning in the department of Antioquia, Colombia | Exclude | No intervention |  |
| Buendia, J. A. et al. | Social and economic variables related with Paraquat self-poisoning: an ecological study | Exclude | No intervention |  |
| Canu, I. G. et al. | Mortality by suicide in the swiss national cohort (1990-2014): Analysis according to occupation and economic activity | Exclude | Wrong outcome |  |
| Carmo, E. A. et al. | Sociodemographic characteristics and time series of mortality due to suicide among elderly individuals in Bahia State, Brazil, 1996-2013 | Exclude | No intervention |  |
| Chacko, B. et al. | Poisoning-the road less travelled | Exclude | Not original research |  |
| Chandra, A. et al. | Paraquat poisoning | Exclude | No intervention |  |
| Chen, X. et al. | Historical trends in suicide risk for the residents of mainland China: APC modeling of the archived national suicide mortality rates during 1987-2012 | Exclude | Wrong outcome |  |
| Chen, Y-Y et al. | The Age-Period-Cohort trends of suicide in Hong Kong and Taiwan, 1979-2018 | Exclude | Wrong outcome |  |
| Chen, Y. Y. et al. | Quantifying the contributions of age, sex, methods, and urbanicity to the changing suicide rate trends in South Korea, 2001-2016 | Exclude | Wrong outcome |  |
| Cruzeiro Szortyka, A. L. S. et al. | Suicidality among South Brazilian tobacco growers | Exclude | Wrong outcome |  |
| Da Cruz Pires, M. C. et al. | Different toxic agents used in suicide attempts in recife. [Portuguese] | Exclude | Selected population |  |
| da Rosa, N. M. et al. | Mortalidade por suicidio no Estado do Parana segundo meios utilizados: Uma analise epidemiologica. [Portuguese] | Exclude | No comparison group |  |
| Damerow, S. M. et al. | Using ex-ante economic evaluation to inform research priorities in pesticide self-poisoning prevention: the case of a shop-based gatekeeper training programme in rural Sri Lanka | Exclude | Wrong outcome |  |
| Dandona, R. et al. | Lessons from a decade of suicide surveillance in India: who, why and how? | Exclude | Wrong outcome |  |
| de Araujo Nascimento, F. et al. | Cultivated areas and rural workers' behavior are responsible for the increase in agricultural intoxications in Brazil? Are these factors associated? | Exclude | No intervention |  |
| De Cassia Dos Santos Nery, T. et al. | Inequalities in pesticide poisoning in brazilian states by ethnicity and race, 2010-2020 | Exclude | No intervention |  |
| Dorooshi, G. et al. | Intoxication-Related Deaths in a Poisoning Center in Isfahan: Demographic and Other-Related Factors | Exclude | No intervention |  |
| dos Santos, A. D. et al | Spatial analysis and temporal trends of suicide mortality in Sergipe, Brazil, 2000-2015 | Exclude | Wrong outcome |  |
| Dos Santos, J. C. P. et al. | Pesticide exposure and poisoning in Brazil: Outcome severity, clinical manifestations and management of cases reported to a poison control center | Exclude | No intervention |  |
| Dos Santos, J. C. P. et al. | Sociodemographic characteristics and exposure patterns of pesticide-related cases reported to a poison service center in Brazil between 2012 and 2016 | Exclude | No intervention |  |
| Duarte, S. K. M. et al. | Temporal trend of mortality by suicide among adults in brazil: 2000 to 2015 | Exclude | Selected population |  |
| Dumitru, M. M. | Characteristics of rural suicide in Romania | Exclude | No intervention |  |
| Eddleston, M. | Poisoning by pesticides | Exclude | Not original research |  |
| Eddleston, M. and Gunnell, D. | Preventing suicide through pesticide regulation | Exclude | Not original research |  |
| Eizadi-Mood, N. et al. | Acute pesticide poisoning in the central part of Iran: A 4-year cross-sectional study | Exclude | Selected population |  |
| Elenga, N. et al. | Clinical features and prognosis of paraquat poisoning in French Guiana: A review of 62 cases | Exclude | Selected population |  |
| Eun, S. J. | Avoidable, amenable, and preventable mortalities in South Korea, 2000-2017: Age-period-cohort trends and impact on life expectancy at birth | Exclude | Wrong outcome |  |
| Feistkorn, E. et al. | Human exposures to pesticides: Results of a subproject of the German pilot study PiMont | Exclude | No comparison group |  |
| Fernando, I. S. et al. | Dramatic improvement of poisoned patient survival in Southern province, Sri Lanka | Exclude | Selected population |  |
| Franck, M. C. et al. | Suicide and associated factors across life span | Exclude | No intervention |  |
| Garcia, E. C. Et al. | Domestic violence and suicide in India | Exclude | Not original research |  |
| Gharbaoui, M. et al. | Pattern of suicide by self-poisoning in Northern Tunisia: An eleven-year study (2005-2015) | Exclude | No intervention |  |
| Ghimire, R. et al. | Trends and methods of suicide in Nepal: A retrospective data analysis | Exclude | No intervention |  |
| Ghodsi, Z. et al. | The mortality rate from self-harm in Iran | Exclude | Wrong outcome |  |
| Gomes, G. A. et al. | Characterization of deaths by suicide between 2013-2017 | Exclude | Wrong outcome |  |
| Gonzalez-Santiago, O. et al. | Unintentional and self-poisoning mortalities in Mexico, 2000-2012 | Exclude | Wrong outcome |  |
| Guarmit, B. et al. | Descriptive epidemiology of suicide attempts and suicide in the remote villages of French Guiana | Exclude | No intervention |  |
| Guseva Canu, I. et al. | Suicide mortality follow-up of the Swiss National Cohort (1990-2014): sex-specific risk estimates by occupational socio-economic group in working-age population | Exclude | Selected population |  |
| Han, D. G. et al. | Suicide Methods According to Age and Sex: An Analysis of Data of 239,565 Suicide Victims in the Republic of Korea From 1991 to 2015 | Exclude | Wrong outcome |  |
| Havassi, N. et al. | Evaluating the predictors of suicide deaths. [Persian] | Exclude | No intervention |  |
| Hendges, C. et al. | Human intoxication by agrochemicals in the region of South Brazil between 1999 and 2014 | Exclude | Wrong outcome |  |
| Holzer, A. et al. | Fatalities due to acute intoxications from 2000 to 2015: A survey of the Poisons Information Centre Austria | Exclude | No intervention |  |
| Jamshidi, F. et al. | Investigation Paraquat Poisoning in Southwest of Iran - from Sign to Mortality and Morbidity | Exclude | Wrong outcome |  |
| Jayaprakash, R. et al. | Fipronil and Acetamiprid Poisoning: New Perils | Exclude | Selected population |  |
| Jeong, K-H and Cho, S. | Gender differences in suicide rates of municipalities in South Korea: A latent profile analysis by life cycles | Exclude | No intervention |  |
| Jiang, H. et al. | Changing of suicide rates in China, 2002-2015 | Exclude | Wrong outcome |  |
| Jors, E. | Pesticide poisonings are not restricted to farmers and vector spray-men! | Exclude | Not original research |  |
| Kamaruzaman, N. A. et al. | Epidemiology and risk factors of pesticide poisoning in Malaysia: a retrospective analysis by the National Poison Centre (NPC) from 2006 to 2015 | Exclude | Wrong outcome |  |
| Kanamori, M. and Kondo, N. | Suicide and Types of Agriculture: A Time-Series Analysis in Japan | Exclude | Wrong outcome |  |
| Karnecki, K. et al. | Epidemiology of suicide in the tri-city metropolitan area in poland in 2010-2019 | Exclude | No intervention |  |
| Karunarathne, A. et al. | How many premature deaths from pesticide suicide have occurred since the agricultural Green Revolution? | Exclude | Not original research |  |
| Kasemy, Z. A. et al. | Incidence, Distribution, and Determinants of Suicide by Self-Poisoning in two Egyptian Provinces | Exclude | Wrong outcome |  |
| Kaur, R. et al. | Study demonstrating pattern of suicidal poisoning deaths in Shaheed Bhagat Singh Nagar (Nawanshahar) of Punjab | Exclude | Wrong outcome |  |
| Khan, A. R. et al. | Suicide and attempted suicide in Jhenaidah District, Bangladesh, 2010-2018 | Exclude | Wrong outcome |  |
| Kidiyoor, A. and Jayprakash, P. | Profile of Poisoning in Autopsy cases in Bangalore South: A Ten-Year Retrospective Study | Exclude | Selected population |  |
| Kim, G-M. et al. | Comparison of the risk factors of Korean adolescent suicide residing in high suicidal regions versus those in low suicidal regions | Exclude | Selected population |  |
| Kim, H. et al. | Predictive Factors Associated With Methods of Suicide: The Korean National Investigations of Suicide Victims (The KNIGHTS Study) | Exclude | No intervention |  |
| Kino, S. et al. | Age, period, cohort trends of suicide in Japan and Korea (1986-2015): A tale of two countries | Exclude | Wrong outcome |  |
| Knipe, D. W. et al. | Regional variation in suicide rates in Sri Lanka between 1955 and 2011: a spatial and temporal analysis | Exclude | Wrong outcome |  |
| Ko, S. et al. | The Burden of Acute Pesticide Poisoning and Pesticide Regulation in Korea | Exclude | Wrong outcome |  |
| Kolves, K. et al. | Choice of a suicide method: Trends and characteristics | Exclude | Wrong outcome |  |
| Kootbodien, T. et al. | Suicide trends by occupation in south africa, 1997 to 2016 | Exclude | Selected population |  |
| Kordrostami, R. et al. | Forensic toxicology analysis of self-poisoning suicidal deaths in Tehran, Iran; trends between 2011-2015 | Exclude | No intervention |  |
| Kramer, D. G. and Ferreira, A. P. | Exogenous poisoning in Tocantins, Northeast Brazil: a retrospective study from 2017 to 2021 | Exclude | Wrong outcome |  |
| Kriengsoontornkij, W. et al. | Patients with toxicological exposures consulted to siriraj poison control center: The analysis of different age groups | Exclude | Selected population |  |
| Kumar, K. et al. | Pesticide poisoning - 10 years retrospective analysis | Exclude | Selected population |  |
| Lapo-Talledo, G. J. et al. | Suicide rates in Ecuador: A nationwide study from 2011 until 2020 | Exclude | No intervention |  |
| Law, Y. W. et al. | Evidence-based suicide prevention: Collective impact of engagement with community stakeholders | Exclude | Wrong outcome |  |
| Lawson, S. | Depression and suicidality in Naivasha, Kenya; investigating the intentionality of poison ingestion and providing clinical tools for those caring for suicidal patients | Exclude | No intervention |  |
| Lekei, E. et al. | Acute pesticide poisoning amongst adolescent girls and women in northern Tanzania | Exclude | Selected population |  |
| Lessa De Souza Maia, G. and Melo Gomes Do Nascimento, M. | Epidemiological profile of the suicidal in the third poorest state of Brazil | Exclude | No intervention |  |
| Lew, B. et al. | Looking into recent suicide rates and trends in Malaysia: A comparative analysis | Exclude | Wrong outcome |  |
| Lin, C-Y et al. | Method-specific suicide rates and accessibility of means: A small-area analysis in Taipei City, Taiwan | Exclude | Wrong outcome |  |
| Liu, Y. et al. | Trends in suicide rates and the case-fatality of pesticide self-poisoning in an agricultural county in china, 2009 to 2014 | Unclear | No intervention |  |
| Liu, Z-R. et al. | Suicide rate trends in China from 2002 to 2015 | Exclude | Wrong outcome |  |
| Magalhaes, A. F. A. and Caldas, E. D. | Underreporting of fatal poisonings in Brazil - A descriptive study using data from four information systems | Exclude | No intervention |  |
| Maheswari, E. et al. | A retrospective analysis on pattern of poisoning and its treatment outcome | Exclude | No intervention |  |
| Maksimovic, Z. M. et al. | Acute organophosphate and carbamate pesticide poisonings - a five-year survey from the National Poison Control Center Of Serbia | Exclude | Selected population |  |
| Marahatta, K. et al. | Suicide burden and prevention in Nepal: The need for a national strategy | Exclude | Not original research |  |
| Martinez-Ales, G. et al. | Age, period, and cohort effects on suicide death in the United States from 1999 to 2018: Moderation by sex, race, and firearm involvement | Exclude | Wrong intervention (e.g. lockable boxes, village stores, manufacturer initiaves) |  |
| Martins, C. C. et al. | Epidemio-toxicological profile of suicide cases: analysis from a forensic unit in Brazil | Exclude | No intervention |  |
| Marupuru, S. et al. | Intentional Self-harm Human Poisoning with Agricultural Micronutrient Foliar Spray: From Rural India of Southern Karnataka | Exclude | Not original research |  |
| Mathur, R. K. et al. | Medico-legal study on the poisoning cases of Ajmer region | Exclude | Selected population |  |
| Matsubayashi, T. and Ueda, M. | Is suicide underreported? Evidence from japan | Exclude | Wrong outcome |  |
| McDonald, K. et al. | Trends in method-specific suicide in Brazil from 2000 to 2017 | Exclude | No intervention |  |
| Miller, C. D. M. and Rudolphi, J. M. | Characteristics of suicide among farmers and ranchers: Using the CDC NVDRS 2003-2018 | Exclude | Selected population |  |
| Mishra, K. K. | Evaluation of attempted suicide cases in farming industry of central maharashtra in comparison to non farming group | Exclude | Selected population |  |
| Mishra, K. K. and Prabhakar, J. V. | Factors influencing suicidal attempt among the agrarian community of central Maharashtra | Exclude | Wrong outcome |  |
| Moradinazar, M. et al. | Estimation of the rate and number of underreported deliberate self-poisoning attempts in western Iran in 2015 | Exclude | Selected population |  |
| Na, J. Y. | Analysis of deaths due to acute intoxication in the republic of korea: An autopsy-based study | Exclude | No intervention |  |
| Nabih, Z. et al. | Epidemiology and risk factors of voluntary pesticide poisoning in Morocco (2008-2014) | Exclude | Wrong outcome |  |
| Naghavi, M. | Global, regional, and national burden of suicide mortality 1990 to 2016: Systematic analysis for the Global Burden of Disease Study 2016 | Exclude | Wrong outcome |  |
| Neves, P. D. M. et al. | Poisoning by agricultural pesticides in the state of Goias, Brazil, 2005-2015: analysis of records in official information systems | Exclude | Wrong outcome |  |
| Nunes, A. M. | Suicide in Portugal: Image of the country. [Portuguese] | Exclude | Wrong outcome |  |
| Nunez-Samudio, V. et al. | Epidemiologic Characteristics of Suicide in Panama, 2007-2016 | Exclude | Wrong outcome |  |
| Okuyama, J. H. H. et al. | Poisoning and associated factors to death from pesticides: case-control study, Brazil, 2017 | Exclude | No intervention |  |
| Ortiz-Prado, E. et al. | The disease burden of suicide in Ecuador, a 15 years' geodemographic cross-sectional study (2001-2015) | Exclude | No intervention |  |
| Osborne, N. J. et al. | Epidemiology of coronial deaths from pesticide ingestion in Australia | Exclude | No intervention |  |
| Pagdhune, A. et al. | Poisoning Cases Reported to Poison Information Centre, Ahmedabad, India: A Three Year Observational Study | Exclude | Selected population |  |
| Page, A. et al. | Suicide by pesticide poisoning remains a priority for suicide prevention in China: Analysis of national mortality trends 2006-2013 | Exclude | No intervention |  |
| Paiman, M. A. and Khan, M. M. | Suicide and deliberate self-harm in Afghanistan | Exclude | Not original research |  |
| Palagani, S. R. et al. | Retrospective Study of Morbidity and Mortality in Organophosphorus Poisoning in and around Vijayawada, Andhra Pradesh | Exclude | Selected population |  |
| Pan, J. et al. | Sharply Reduced but Still Heavy Self-Harm Burdens in Hubei Province, China, 1990-2015 | Exclude | No intervention |  |
| Panzoo, Q. R. et al. | Assessment of pattern of suicidal, accidental and homicidal cases along with the sort of poisoning in north Indian population | Exclude | No intervention |  |
| Parekh, U. and Gupta, S. | Epidemio-toxicological profile of poisoning cases - A five years retrospective study | Exclude | Selected population |  |
| Pedersen, B. et al. | Characteristics of Pesticide Poisoning in Rural and Urban Settings in Uganda | Exclude | Selected population |  |
| Peshin, S. S. and Gupta, Y. K. | Poisoning due to household products: A ten years retrospective analysis of telephone calls to the National Poisons Information Centre, All India Institute of Medical Sciences, New Delhi, India | Exclude | Wrong outcome |  |
| Pinto, L. L. T. et al. | Mortality trend due to intentionally self-inflicted injuries in Brazil from 2004 to 2014 | Exclude | Wrong outcome |  |
| Pompili, M. | Suicide prevention and access to lethal methods | Exclude | Not original research |  |
| Prakruthi, N. et al. | Pesticide self poisoning: A study of suicidal intent, psychiatric morbidity and access to pesticides | Exclude | Selected population |  |
| Qiao, J. et al. | The reversing trend in suicide rates in Shanghai, China, from 2002 to 2020 | Exclude | No comparison group |  |
| Qin et al. | The waterfall pattern of suicide mortality in Inner Mongolia for 2008-2015 | Include |  |  |
| Queiroz, P. R. et al. | Notifiable Diseases Information System and human poisoning by pesticides in Brazil | Exclude | Wrong outcome |  |
| Rajapakse, T. et al. | The Impact of the COVID-19 Pandemic and Lockdowns on Self-Poisoning and Suicide in Sri Lanka: An Interrupted Time Series Analysis | Exclude | No comparison group |  |
| Rappai, Rija et al. | Suicide research in India: An overview of four decades | Exclude | Not original research |  |
| Ravindra Kumar, C. N. et al. | Profile of deaths due to poisoning at a tertiary care centre in north karnataka | Exclude | Selected population |  |
| Razwiedani, L. L. and Rautenbach, P. | Epidemiology of Organophosphate Poisoning in the Tshwane District of South Africa | Exclude | No intervention |  |
| Rosa, N. M. Et al. | Suicide mortality according to methods used in Parana State: An epidemiological analysis | Exclude |  |  |
| Rother, H. A. | Pesticide suicides: what more evidence is needed to ban highly hazardous pesticides? | Exclude | Not original research |  |
| Safdar, M. et al. | Suicide by poisoning in Pakistan: review of regional trends, toxicity and management of commonly used agents in the past three decades | Exclude | Not original research |  |
| Sarkar, S. | Despite historic bans, south Asia still struggles with pesticide suicides | Exclude | Not original research |  |
| Settimi, L. et al. | Cases of human exposure identified in 2015-2018 by the Italian surveillance system of toxic exposures and poisonings | Exclude | No intervention |  |
| Sharma, R. et al. | Mortality and morbidity associated with acute poisoning cases in north-east India: A retrospective study | Exclude | Selected population |  |
| Shetty, A. R. S. et al. | Pattern of poisoning in autopsy cases at a tertiary care center in bengaluru - A five year retrospective study | Exclude | Selected population |  |
| Shrivastava, S. R. et al. | Limiting pesticide access to minimize suicide incidence | Exclude | Not original research |  |
| Singh, B. et al. | Epidemiological profile of complete suicidal poisoning cases autopsied at autopsy centre, RIMS, Ranchi | Exclude | Wrong outcome |  |
| Snowdon, J. | Differences between patterns of suicide in East Asia and the West. The importance of sociocultural factors | Exclude | Not original research |  |
| Snowdon, J. et al. | Changes in age patterns of suicide in Australia, the United States, Japan and Hong Kong | Exclude | Wrong outcome |  |
| Sohn, K. | The trend in suicide methods in South Korea in 1997-2015 | Unclear | Wrong outcome |  |
| Stuart, A. M. et al. | Agriculture without paraquat is feasible without loss of productivity-lessons learned from phasing out a highly hazardous herbicide | Exclude | Not original research |  |
| Sturer, A. et al. | National poisons centre data collection: Pilot study on Pesticide Poisoning Monitoring in Germany (PIMONT-PES) | Exclude | No intervention |  |
| Suanrueang, P. et al. | Gender Differences in Committing Suicide in Thailand | Exclude | Wrong outcome |  |
| Sumner, A. E. et al. | Socioeconomic, psychosocial, and healthcare-access contributors to poisoning and suicide in sri lanka: An ecological survey | Exclude | No intervention |  |
| The Lancet (editorial) | Phasing out harmful use of pesticides | Exclude | Not original research |  |
| Thippesh Kumar, N. et al. | A study of epidemiology of poisoning cases brought for autopsy at tertiary health care centre in south-western maharashtra: A ten-year retrospective study | Exclude | Selected population |  |
| Tong, Y. et al. | Relationship of the high proportion of suicidal acts involving ingestion of pesticides to the low male-to-female ratio of suicide rates in China | Exclude | No intervention |  |
| Tu, C. Y. et al. | Characteristics and psychopathology of 1,086 patients who self-poisoned using pesticides in Taiwan (2012-2019): A comparison across pesticide groups | Exclude | No intervention |  |
| Ullah, I. et al. | Suicide in low- and middle-income countries: Perspectives form overview of studies in South Asia | Exclude | Not original research |  |
| Utyasheva, L. and Eddleston, M. | Removing highly hazardous pesticides from Indian agriculture will reduce suicides | Exclude | Not original research |  |
| Vapa, D. et al. | Analysis of Suicides in the Province of Vojvodina, Republic of Serbia, during the 2001-2015 Period | Exclude | No intervention |  |
| Velasquez, I. M. et al. | Suicide trends and self-harm in Panama: Results from the National Mortality Registry and hospital-based data | Exclude | No intervention |  |
| Velazquez-Vazquez, D. et al. | Epidemiological description of suicide mortality in the state of Yucatan between 2013 and 2016 | Exclude | No intervention |  |
| Vijayakumar, L. | Challenges and opportunities in suicide prevention in South-East Asia | Exclude | Not original research |  |
| Vijayakumar, L. et al. | Suicide Prevention in the Southeast Asia Region | Exclude | Not original research |  |
| Vipparla, K. R. and Vijaya Kumari, N. | Current trends of poisoning in tertiary care hospitals located in a rural area of Salem, Tamil Nadu, India | Exclude | Selected population |  |
| Wang, B. et al. | Self-poisoning with pesticides in Jiangsu Province, China: a cross-sectional study on 24,602 subjects | Exclude | No intervention |  |
| Wang, L. et al. | Poisoning deaths in China, 2006-2016 | Exclude | Wrong outcome |  |
| Wang, L. L. et al. | A retrospective study of poisoning deaths from forensic autopsy cases in northeast China (Liaoning) | Exclude | Wrong outcome |  |
| Wang, N. et al. | [Epidemiological characteristics of pesticide poisoning in Xuzhou city from 2005 to 2017] | Exclude | No comparison group |  |
| Weerasinghe, M. et al. | Gatekeeper training for vendors to reduce pesticide self-poisoning in rural South Asia: a study protocol for a stepped-wedge cluster randomised controlled trial | Exclude | Not original research |  |
| Woyessa, A. H. and Palanichamy, T. | Patterns, Associated Factors, and Clinical Outcomes of Poisoning among Poisoning Cases Presented to Selected Hospitals in Western Ethiopia: Hospital-Based Study | Exclude | Selected population |  |
| Wu, Y. et al. | Sex-specific and age-specific suicide mortality by method in 58 countries between 2000 and 2015 | Exclude | No comparison group |  |
| Xiao, L. et al. | A 9-year retrospective study of poisoning-related deaths in Southwest China (Sichuan) | Exclude | Wrong outcome |  |
| Yeum, T-S. et al. | Factors affecting suicide method lethality among suicide attempters in the Korea National Suicide Survey | Exclude | Selected population |  |
| Zdarova Karasova, J. | Toxic effects of pesticides. [Czech] | Exclude | Wrong outcome |  |
| Zhao, G. et al. | Farmer's suicide with the use of pesticides in different areas in Spain | Exclude | No intervention |  |
| Zheng, X. W. et al. | [Analysis of spatial-temporal distribution of pesticide poisoning in Quzhou, 2013-2017] | Exclude | Wrong outcome |  |
| Zhu, R. T. et al.. | Suicide Means Used by the Older Adults in Rural China: A Comparison Between Those Using Pesticides and Other Means | Exclude | Selected population |  |
| **New publications** | | | | |
| Chan, L. F. et al. | Surveillance of pesticide posioning in an East and a West Malaysian hospital: characteristics of pesticide poisoning and the early impact of a national Paraquat ban | Exclude | Selected population |  |
| Moller, J. et al. | Trends in intentional and unintentional posionings among older adults - a national register-based study in Sweden | Exclude | Wrong outcome |  |
| Yan, Y. et al. | Impact of pesticide regulations on mortality from suicide by pesticide in China: an interrupted time series analysis | Include |  |  |
| **Personal library collection** | | | | |
| Lin, C-Y et al. | Impact of the paraquat ban on reducing suicide in Taiwan: the effect on 2020 suicide rates | Include |  |  |
| Chan, L. F. et al. | Surveillance of pesticide poisoning in an East and West Malaysian hospital: characteristics of pesticide posioning and the early impact of national Paraquat ban | Exclude | Selected population |  |
| Kim, J. W. et al. | Paraquat: toxicology and impacts of its ban on human health and agriculture | Exclude | Not original research |  |
