# Supplementary material for: Preventing suicide by restricting access to Highly Hazardous Pesticides (HHPs): A systematic review of international evidence since 2017
Source: PLOS Glob Public Health. 2025 Feb 3;5(2):e0003785. doi: 10.1371/journal.pgph.0003785 (PMC11790168; doi:10.1371/journal.pgph.0003785)
Supplement: S2 Table — (DOCX) [file pgph.0003785.s004.docx]

**Supplementary materials**

**S2 Table.** Study main characteristics

| Country (main author), data extractors (date) | Year of data collection | Ban level | Pesticides banned | Proportion of suicide due to pesticides | Potential confounding variables and historic events during study period |
| --- | --- | --- | --- | --- | --- |
| Low- and middle-income countries | | | | |  |
| China (Yan) (1), BR (2023 Oct 20), CY (2023 Sep 14) | 2006 to 2018 | National | 5 pesticides: methamidophos, parathion-methyl, parathion, monocrotophos, ammonium phosphate) in 2008  New paraquat parent drug in 2012  Paraquat aqueous solution in 2016 | 51.2% in 2006, decreased to 36.8% in 2018 | **No visible effect-**  Examined trends by age group, sex, and urbanization level, which is associated with SEP, heath status and migration patterns in China. Natural variation was discounted by comparing urban to rural areas and sex-specific rates. |
| India (Arya) (2), BR (2023 Aug 25), CY (2023 Sep 14) | 2001 to 2014 | National | Endosulfan in 2011 | Not reported | **Not assessed** |
| India (Bonvoisin) (3),  BR (2023 Aug 18), CY (2023 Sep 14) | 1995 to 2015 | National | Endosulfan in 2011 | 18% between 1995 and 2015 | **No visible effect-**  Examined unemployment trends and GDP per capita. |
|  |  | Regional (Kerala) | Endosulfan in 2005 |  |  |
|  |  |  | 14 pesticides in 2011: anilofos, atrazine, carbofuran, edifenphos, methoxy ethyl mercuric chloride, methyl parathion, monocrotophos, oxythioquinox, paraquat, phorate, profenofos, thiobencarb, triazophos, tricyclazole |  |  |
| Mongolia (Qin) (4), BR (2023 Oct 20), CY (2023 Sep 15) | Jan 2008 to Dec 2015 | Regional (Inner Mongolia) | 2 pesticides in 2008: Octachlorodipropyl ether, Fiponel | 47.94% in 2008-2011, and 36.36% in 2012-2015 | **Potential effect in reducing suicide rates-**  Series of social policies/interventions to improve living standards and reduce poverty, which benefited low-income population was implemented in 2012: poverty alleviating policies and investment on education funds (by 50%) elevated 13.4% of the population in Inner Mongolia out of poverty. |
|  |  |  | 10 organophosphorus pesticides in 2011: Fenamiphos, Phosfolan-methyl, Fonofos, Calcium phosphide, Magnesium phosphide, Zinc phosphide, Cadusafos, Coralox, Sulfotep, Terbufos |  |  |
|  |  |  | 2 pesticides in 2012: Paraquat, Chlorsulfuron |  |  |
| High income countries | | | | |  |
| Japan (Eddleston) (5), BR (2023 Sep 27), CY (2023 Sep 15) | 1966 to 2019 | National | Paraquat | 8.6% in 1985 | **Not assessed** |
| South Korea (Cha) (6), BR (2023 Sep 20), CY (2023 Sep 15) | 1991 to 2014 | National | Paraquat | 21% between 2006 and 2010 | **Adjusted for in models-**  Examined % of people involved in farming, and unemployment, dependency and divorce rates. Also considered the effect of the 1997 Asian economic crisis and the 2008 Great Recession (as dummy variables in the models). |
| South Korea (Kim) (7), BR (2023 Oct 4), CY (2023 Sep 15) | 2009 to 2013 | National | Paraquat | 9.7% of deaths from external causes before the intervention | **Potential small effect, direction unclear -** Examined gender, age groups, residence, occupation, marital status, and education level. Proportion of divorce and unemployed slightly decreased in post-intervention period; however, proportion of non-metropolis slightly increased. |
| Taiwan (Chang) (8), BR (2023 Sep 20), CY (2023 Sep 15) | 2011 to 2019 | National | Paraquat | 12.1% between 2011 and 2017 | **Adjusted for in models (interaction terms)-** Area (urban or rural based on proportion of agricultural workers), year, sex, and age group.  Also examined the impact of the intervention on paraquat suicides specifically. |
| Taiwan (Lin) (9), CY (2023 Sep 15) | 2011 to 2020 | National | Paraquat | 12.1% between 2011 and 2017 | **Adjusted for in models-** Sex, age group, and year. |

**References**

1. Yan Y, Jiang Y, Liu R, Eddleston M, Tao C, Page A, et al. Impact of pesticide regulations on mortality from suicide by pesticide in China: an interrupted time series analysis. Front Psychiatry. 2023;14:1189923.

2. Arya V, Page A, Gunnell D, Armstrong G. Changes in method specific suicide following a national pesticide ban in India (2011–2014). J Affect Disord. 2021;278:592-600.

3. Bonvoisin T, Utyasheva L, Knipe D, Gunnell D, Eddleston M. Suicide by pesticide poisoning in India: a review of pesticide regulations and their impact on suicide trends. BMC Public Health. 2020;20(1):251.

4. Qin P, Du M, Wang S, Zhang X, Wang Y, Yan T, et al. The waterfall pattern of suicide mortality in Inner Mongolia for 2008–2015. J Affect Disord. 2019;256:331-6.

5. Eddleston M, Nagami H, Lin CY, Davis ML, Chang SS. Pesticide use, agricultural outputs, and pesticide poisoning deaths in Japan. Clin Toxicol (Phila). 2022;60(8):933-41.

6. Cha ES, Chang SS, Choi Y, Lee WJ. Trends in pesticide suicide in South Korea, 1983-2014. Epidemiol Psychiatr Sci. 2019;29:e25.

7. Kim J, Shin SD, Jeong S, Suh GJ, Kwak YH. Effect of prohibiting the use of Paraquat on pesticide-associated mortality. BMC Public Health. 2017;17(1):858.

8. Chang SS, Lin CY, Lee MB, Shen LJ, Gunnell D, Eddleston M. The early impact of paraquat ban on suicide in Taiwan. Clin Toxicol (Phila). 2022;60(1):131-5.

9. Lin CY, Hsu CY, Lee M-B, Chang SS. Impact of the paraquat ban on reducing suicide in Taiwan: the effect on 2020 suicide rates. J Suicidology. 2022;17(1):80-7.
