# Supplementary material for: Preventing suicide by restricting access to Highly Hazardous Pesticides (HHPs): A systematic review of international evidence since 2017
Source: PLOS Glob Public Health. 2025 Feb 3;5(2):e0003785. doi: 10.1371/journal.pgph.0003785 (PMC11790168; doi:10.1371/journal.pgph.0003785)
Supplement: S3 Table — (DOCX) [file pgph.0003785.s005.docx]

**Supplementary materials**

**Table S3.** Additional information on type of regulation change, their effect on pesticide suicide and overall suicides, and methods applied for data analyses.

| Country (main author, year) | Type of regulation change | Analyses method | Effect on pesticide suicides | Effect on overall suicides |
| --- | --- | --- | --- | --- |
| Low- and middle-income countries | | | | |
| China (Yan, 2023) (1) | Intervention 1: ban of 5 pesticides (methamidophos, parathion-methyl, parathion, monocrotophos, ammonium phosphate) in December 2008. This ban prohibited domestic production, circulating, and using on their own or mixed with other substances  Intervention 2: banning registration and production of new paraquat parent drug and aqueous solutions, and regulating the existing paraquat production and after-sales services in April 2012  Intervention 3: banning domestic sales and use of paraquat aqueous solution in July 2016 | Interrupted time series regression model using segmented regression analysis and GLS method. They adjusted for seasonal variations using Fourier terms consisting of sine/cosine three pairs. Reported rate ratios (RR) and 95% CI for underlying trend, and level and trend change after each intervention. | **Decreased –**  The age-standardised pesticide suicide rate decreased by 60.5%, from 6.50 in 2006 to 2.56 per 100,000 in 2018. | **Decreased –**  The age-standardised overall suicide decreased by 45.1%, from 12.7 in 2006 to 6.98 per 100,000 people in 2018. |
| India (Arya, 2021) (2) | National ban on endosulfan in May 2011 | Difference between observed and expected rates of suicides were calculated. Piecewise linear regression models for different suicide methods, with year of intervention (2011) specified a priori. Reports standardised beta coefficient, 95% CI. | **Decreased** –  For males:  The rate difference between observed and expected insecticide poisoning was -1.27 per 100,000 in 2014.  The standardised beta coefficient (95% CI) for insecticide suicide in males was -0.02 (95%CI -0.05 to 0.01) for the 2001 -2010 period and -0.3 (-0.48 to -0.22) for the 2011-2014 period.  For females:  The rate difference between observed and expected insecticide poisoning was -0.63 per 100,000 in 2014.  The standardised beta coefficient (95% CI) for insecticide suicide in females was -0.02 (95%CI -0.04 to -0.01) for the 2001 -2010 period and -0.19 (-0.25 to -0.13) for the 2011-2014 period. | **Not reported** |
| India (Bonvoisin, 2020) (3) | National ban on domestic production of endosulfan in May 2011; finals stocks had to be disposed of or exported by January 2017 | Negative binomial regressions for suicide rates after bans compared to those predicted based on pre-ban trends. Rate ratios (with 95% CI) were calculated for each year after the ban and compared with predicted rates based on extrapolated trends before each ban. | **Decreased** –  Rate ratios of observed pesticide suicide rate were 0.83 (95% 0.81 to 0.86) in 2011, 0.75 (95% 0.72 to 0.77) in 2012, 0.71 (95% 0.69 to 0.74) in 2013, and 0.52 (95% 0.47 to 0.54) in 2014. | **Decreased** –  Rete ratios of observed overall suicide rate were 1.00 (95% CI 0.98 to 1.02) in 2011, 0.97 (95% CI 0.95 to 1.00) in 2012, 0.95 (95% CI 0.92 to 0.97) in 2013, and 0.90 (95% CI 0.87 to 0.93) in 2014. |
|  | Regional ban on endosulfan in Kerala in October 2005;  Regional ban on 14 pesticides (anilofos, atrazine, carbofuran, edifenphos, methoxy ethyl mercuric chloride, methyl parathion, monocrotophos, oxythioquinox, paraquat, phorate, profenofos, thiobencarb, triazophos, tricyclazole) in Kerala in January 2011 |  | **Decreased –**  After the 2005 ban on endosulfan, the rate ratios of observed pesticide suicide rate were 0.87 (95% CI 0.77 to 0.99) in 2006, 0.77 (95% CI 0.66 to 0.89) in 2007, 0.83 (95% CI 0.70 to 0.99) in 2008, 0.83 (95% CI 0.68 to 1.01) in 2009, and 0.79 (95% CI 0.64 to 0.99) in 2010.  After the 2011 ban on 14 pesticides, the rate ratios of observed pesticide suicide rate were 0.85 (95% CI 0.80 to 0.91) in 2011, 0.73 (95% CI 0.68 to 0.78) in 2012, 0.51 (95% CI 0.47 to 0.55) in 2013, and 0.45 (95% CI 0.42 to 0.49) in 2014. | **Increased -**  After the 2005 ban on endosulfan, rate ratios of observed overall suicide rate were 0.99 (95% CI 0.96 to 1.02) in 2006 and 2007, 0.95 (95% CI 0.92 to 0.99) in 2008, 0.98 (95% CI 0.94 to 1.03) in 2009, and 0.97 (95% CI 0.93 to 1.02) in 2010.  After the 2011 ban on 14 pesticides, the rate ratios of observed pesticide suicide rate were 0.99 (95% CI 0.98 to 1.01) in 2011, 1.01 (95% CI 0.99 to 1.03) in 2012, 1.04 (95% CI 1.01 to 1.06) in 2013, and 1.02 (95% CI 1.00 to 1.05) in 2014. |
| Mongolia (Qin, 2019) (4) | Ban of 2 pesticides (octachlorodipropyl ether and fiponel) in 2008 | Used Chi-square tests to compare the suicide mortality between the two periods (2008-2011 and 2012-2015). Logistic regression models to assess the risk of suicide between the two periods by gender and region. Reported OR and 95% CI. | **Decreased –**  The odds ratio for pesticide poisoning was higher (1.98, 95% CI 1.702 to 2.310) in the 2008-2011 period compared to the 2012-2015 period. | **Decreased –**  Overall suicide mortality was higher in 2008-2011 (7.20 per 100,000) compared to 2012-2015 (4.84 per 100,000) (X^2^ = 62.28, P = 0.00). |
|  | Ban of 10 organophosphorus pesticides (fenamiphos, phosfolan-methyl, fonofos, calcium phosphide, magnesium phosphide, zinc phosphide, cadusafos, coralox, sulfotep, terbufos) in 31 October 2011 by the Ministry of Agriculture |  |  |  |
|  | Ban of 2 pesticides (paraquat and chlorsulfuron) in 2012 |  |  |  |
| High-income countries | | | | |
| Japan (Eddleston, 2022) (5) | Paraquat was registered in Japan in 1962 as a liquid SL20 formulation. Restrictions on sale and use of this formulation were imposed in 1986 by the Ministry of Agriculture, Forestry and Fisheries and a 4.3% paraquat ion + 4.1% diquat ion combination product was registered in its place. | Linear regression using Prais-Winsten estimation to account for first-order autocorrelation in time-series data. The models were adjusted for year and unemployment rate. However, these models were only used to assess relationship between pesticide sales volume and mortality. | **Decreased –**  Number of pesticide suicides decreased from 2,038 (8.6% of all suicides) in 1985 to 146 (0.7% of all suicides) in 2019. | **Not reported** |
| South Korea (Cha, 2020) (6) | Re-registration of paraquat was cancelled from the end of November 2011 and its sale was completely banned from the end of October 2012. | Multivariable linear regression models using Prais-Winsten estimation to account for first-order autocorrelation. | **Decreased –**  Annual percentage change from 2001 to 2014 was -28.2% (95%CI -36.7 to -18.6) following the paraquat ban in 2011-2012.  Coefficients of the association between paraquat ban and pesticide suicide rate in 1983-2014: -1.32 (95% CI: -3.03 to 0.40; adjusted for calendar year only) and -1.47 (95% CI -3.22 to 0.29; adjusted for the amount of pesticides sold, the proportion of people involved in farming, unemployment rate, dependency ratio, divorce rate, the 1997 Asian economic crisis, and the 2008 Great Recession) | **Not reported –**  However, figure 1S (supplementary) shows a continuation of the upwards trend in non-pesticide suicides as pesticide suicide rates declined from 2003/2004. |
| South Korea (Kim, 2017) (7) | Re-registration of paraquat was cancelled from the end of November 2011 and its sale was completely banned from the end of October 2012. | Multivariable logistic regression, adjusted for age, gender, residence, season when death occurred, weekday when death occurred, occupation, marital status, education level, and death intent. An interaction term was added to examine the interaction between intervention and intent group. Reported adjusted OR, with 95% CI. | **Decreased –**  Pesticide poisoning deaths decreased by approximately 50%, from 150 in October 2012 to approximately 75 in December 2013 (extrapolated from figure 2).  Pesticide-associated suicide mortality after the ban decreasd by 39% (95% CI 36% to 42%) compared to that before the ban (2009-2010) after adjusting for gender, age, season, weekday, residence, job, marital status, education level, intent, and interaction term (intervention*intent) in the multivariable logistic regression. | **Not reported** |
| Taiwan (Chang, 2022) (8) | Nationwide first-stage ban on the import and production of Paraquat from February 2018. This was followed by a complete second-stage ban on its sale and use from February 2020, but no post-ban data presented. | Negative binomial regression models, adjusted for year, sex, and age groups. Reported estimate rate ratios (RR) and 95% CI in 2018 and 2019 compared with the expected suicide rates based on the pre-ban linear suicide trends (2011-2017). | **Decreased –**  Pesticide suicide rates decreased by 37% (RR = 0.63, 95%CI 0.54 to 0.74) in 2019, which corresponded to an estimated rate reduction of 0.93 (95% CI 0.57 to 1.35) per 100,000 or 190 (95% CI 116 to 277) in number of suicides. This decrease was mainly attributed to the reduction in paraquat suicides (RR = 0.42, 95% CI 0.33 to 0.54, a rate reduction of 0.71 (95% CI 0.45 to 1.04) per 100,000 and 145 (95% CI 92 to 213) fewer suicides. Non-paraquat pesticide suicides had a small reduction (RR = 0.82, 95 %CI 0.67 to 1.00; rate difference = -0.23 per 100,000, 95% CI -0.51 to 0.01). | **No change –**  No evidence of statistically significant change in overall suicides in 2019 (RR = 1.00, 95% CI 0.95 to 1.06). |
| Taiwan (Lin, 2022) (9) | Nationwide first-stage ban on the import and production of Paraquat from February 2018. This was followed by a complete second-stage ban on its sale and use from February 2020, but no post-ban data presented. | Negative binomial regression models. Reported estimate rate ratios (RR) and 95% CI. | **Decreased –**  Pesticide suicide rates decreased by 44% (RR = 0.56, 95% CI 0.47 to 0.67) in 2020. The corresponding estimated reduced number in pesticide suicides was 225 (95% CI 128 – 284) and rate of pesticide suicides was 1.10 (95% CI 0.69 to 1.59) per 100,000. Paraquat suicides decreased by 74% (RR = 0.26, 95% CI 0.19 to 0.34), and the rate difference was -0.95 (95% CI -1.39 to -0.62) per 100,000, with 195 (95% CI 128 to 284) fewer suicides. Non-paraquat pesticide suicides had a marginal reduction (RR = 0.86, 95 %CI 0.69 to 1.08; rate difference = -0.17 per 100,000, 95% CI -0.49 to 0.09). | **Decreased –**  Overall suicide rates decreased 7% (RR = 0.93, 95%CI 0.87 to 0.99) per 100,000 in 2020. |

**References**

1. Yan Y, Jiang Y, Liu R, Eddleston M, Tao C, Page A, et al. Impact of pesticide regulations on mortality from suicide by pesticide in China: an interrupted time series analysis. Front Psychiatry. 2023;14:1189923.

2. Arya V, Page A, Gunnell D, Armstrong G. Changes in method specific suicide following a national pesticide ban in India (2011–2014). J Affect Disord. 2021;278:592-600.

3. Bonvoisin T, Utyasheva L, Knipe D, Gunnell D, Eddleston M. Suicide by pesticide poisoning in India: a review of pesticide regulations and their impact on suicide trends. BMC Public Health. 2020;20(1):251.

4. Qin P, Du M, Wang S, Zhang X, Wang Y, Yan T, et al. The waterfall pattern of suicide mortality in Inner Mongolia for 2008–2015. J Affect Disord. 2019;256:331-6.

5. Eddleston M, Nagami H, Lin CY, Davis ML, Chang SS. Pesticide use, agricultural outputs, and pesticide poisoning deaths in Japan. Clin Toxicol (Phila). 2022;60(8):933-41.

6. Cha ES, Chang SS, Choi Y, Lee WJ. Trends in pesticide suicide in South Korea, 1983-2014. Epidemiol Psychiatr Sci. 2019;29:e25.

7. Kim J, Shin SD, Jeong S, Suh GJ, Kwak YH. Effect of prohibiting the use of Paraquat on pesticide-associated mortality. BMC Public Health. 2017;17(1):858.

8. Chang SS, Lin CY, Lee MB, Shen LJ, Gunnell D, Eddleston M. The early impact of paraquat ban on suicide in Taiwan. Clin Toxicol (Phila). 2022;60(1):131-5.

9. Lin CY, Hsu CY, Lee M-B, Chang SS. Impact of the paraquat ban on reducing suicide in Taiwan: the effect on 2020 suicide rates. J Suicidology. 2022;17(1):80-7.
