# Supplementary material for: Preventing suicide by restricting access to Highly Hazardous Pesticides (HHPs): A systematic review of international evidence since 2017
Source: PLOS Glob Public Health. 2025 Feb 3;5(2):e0003785. doi: 10.1371/journal.pgph.0003785 (PMC11790168; doi:10.1371/journal.pgph.0003785)
Supplement: Attachment — Submitted filename: Rebuttal letter_Plos Global Public Health.docx [file pgph.0003785.s007.docx]

Journal Requirements:

1. Please review your reference list to ensure that it is complete and correct. If you have cited papers that have been retracted, please include the rationale for doing so in the manuscript text, or remove these references and replace them with relevant current references. Any changes to the reference list should be mentioned in the rebuttal letter that accompanies your revised manuscript. If you need to cite a retracted article, indicate the article’s retracted status in the References list and also include a citation and full reference for the retraction notice.

We have checked all the references to confirm that they are up-to-date and do not refer to manuscripts that have been retracted.

Additional Editor Comments (if provided):

Thank you very much for the revised manuscript. Reviewer #1 recommends minor revisions to more accurately frame this review article: "all references made in the manuscript that this would be an ‘updated’ systematic review should be revised. In the title, “an updated systematic review” should be changed to “a systematic review of international evidence since 2017”. This is an important point. Same for the abstract, where it should be explicitly mentioned that the review only includes data from 2017 onwards (both in the ‘methods’ [for search strategy] and ‘interpretation’ [as a limitation] sections of the abstract). This should also be discussed in the discussion section of the manuscript."

We thank the editor for highlighting Reviewer #1’s requests and we can confirm that we have made all the changes suggested by them to clarify that this is a systematic review from 2017 onwards. We have revised any mentions of an update of the previous review from the main text and have discussed this in the discussion section of the manuscript.

[Note: HTML markup is below. Please do not edit.]

Reviewers' comments:

Reviewer's Responses to Questions

**Comments to the Author**

1. If the authors have adequately addressed your comments raised in a previous round of review and you feel that this manuscript is now acceptable for publication, you may indicate that here to bypass the “Comments to the Author” section, enter your conflict of interest statement in the “Confidential to Editor” section, and submit your "Accept" recommendation.

Reviewer #1: (No Response)

Reviewer #2: All comments have been addressed

2. Does this manuscript meet PLOS Global Public Health’s [publication criteria](https://c05y1x9s.r.us-east-2.awstrack.me/L0/https:%2F%2Fjournals.plos.org%2Fglobalpublichealth%2Fs%2Fcriteria-for-publication/1/010f01929584fa4b-74190115-5d13-4d18-8b63-33f81afd3e05-000000/xayO1-4uxl3cccbeSX6pg2yJLbA=180)? Is the manuscript technically sound, and do the data support the conclusions? The manuscript must describe methodologically and ethically rigorous research with conclusions that are appropriately drawn based on the data presented.

Reviewer #1: (No Response)

Reviewer #2: (No Response)

3. Has the statistical analysis been performed appropriately and rigorously?

Reviewer #1: (No Response)

Reviewer #2: (No Response)

4. Have the authors made all data underlying the findings in their manuscript fully available (please refer to the Data Availability Statement at the start of the manuscript PDF file)?

The [PLOS Data policy](https://c05y1x9s.r.us-east-2.awstrack.me/L0/https:%2F%2Fjournals.plos.org%2Fglobalpublichealth%2Fs%2Fdata-availability/1/010f01929584fa4b-74190115-5d13-4d18-8b63-33f81afd3e05-000000/KiOrXTj0BN5s98o7aTM_qg4qs7g=180) requires authors to make all data underlying the findings described in their manuscript fully available without restriction, with rare exception. The data should be provided as part of the manuscript or its supporting information, or deposited to a public repository. For example, in addition to summary statistics, the data points behind means, medians and variance measures should be available. If there are restrictions on publicly sharing data—e.g. participant privacy or use of data from a third party—those must be specified.

Reviewer #1: (No Response)

Reviewer #2: (No Response)

5. Is the manuscript presented in an intelligible fashion and written in standard English?

PLOS Global Public Health does not copyedit accepted manuscripts, so the language in submitted articles must be clear, correct, and unambiguous. Any typographical or grammatical errors should be corrected at revision, so please note any specific errors here.

Reviewer #1: (No Response)

Reviewer #2: (No Response)

6. Review Comments to the Author

Please use the space provided to explain your answers to the questions above. You may also include additional comments for the author, including concerns about dual publication, research ethics, or publication ethics. (Please upload your review as an attachment if it exceeds 20,000 characters)

Reviewer #1: My main concern with the previous version of the manuscript was the fact that this was not an ‘updated review’ but merely a (minor) sequel of the previous review conducted by several of the same authors.

I’m afraid that simply adding a short summary of the 2017 review (section ‘Summary of studies reported by Gunnell et al’) in their revision (still) does not make this an ‘updated’ systematic review.

By only including nine studies published from 2017 onwards, this manuscript still provides a very selective overview of the evidence as it omits data from many non-Asian countries (eg, Denmark, Finland, Germany, USA, Colombia, Jordan) that were identified in the 2017 Gunnell review. The short summary provided in the manuscript does not address this limitation.

Therefore, all references made in the manuscript that this would be an ‘updated’ systematic review should be revised. In the title, “an updated systematic review” should be changed to “a systematic review of international evidence since 2017”. This is an important point. Same for the abstract, where it should be explicitly mentioned that the review only includes data from 2017 onwards (both in the ‘methods’ [for search strategy] and ‘interpretation’ [as a limitation] sections of the abstract). This should also be discussed in the discussion section of the manuscript.

We have revised our manuscript according to the suggestions by the reviewer. We have deleted any mentions of an update of the previous review from the main text and have emphasised that this is a review including data from 2017 onwards. We have also discussed this in the discussion section of the manuscript.

Reviewer #2: (No Response)

7. PLOS authors have the option to publish the peer review history of their article ([what does this mean?](https://c05y1x9s.r.us-east-2.awstrack.me/L0/https:%2F%2Fjournals.plos.org%2Fglobalpublichealth%2Fs%2Feditorial-and-peer-review-process%23loc-peer-review-history/1/010f01929584fa4b-74190115-5d13-4d18-8b63-33f81afd3e05-000000/mr0hQ7laf5UP2nZOeO_DqLQN848=180)). If published, this will include your full peer review and any attached files.


**Do you want your identity to be public for this peer review?** If you choose “no”, your identity will remain anonymous but your review may still be made public.


For information about this choice, including consent withdrawal, please see our [Privacy Policy](https://c05y1x9s.r.us-east-2.awstrack.me/L0/https:%2F%2Fwww.plos.org%2Fprivacy-policy/1/010f01929584fa4b-74190115-5d13-4d18-8b63-33f81afd3e05-000000/Vz_Ssdg_E4_nwWysVAzL4v5QOGI=180).

Reviewer #1: No

Reviewer #2: No
