# Supplementary material for: Preventing suicide by restricting access to Highly Hazardous Pesticides (HHPs): A systematic review of international evidence since 2017
Source: PLOS Glob Public Health. 2025 Feb 3;5(2):e0003785. doi: 10.1371/journal.pgph.0003785 (PMC11790168; doi:10.1371/journal.pgph.0003785)
Supplement: Attachment — Submitted filename: Point by point reply to reveiwers_Plos Global Public Health.docx [file pgph.0003785.s006.docx]

Thank you for taking the time to review our manuscript and to provide feedback. We very much appreciate it and have edited the manuscript following the suggestions provided by the reviewers. Please find our point-by-point reply below (in blue).

Additional Editor Comments (if provided):

Thank you very much for submitting this manuscript to PLOS Global Public Health. Highly hazardous pesticides represent an important global public health issue. Both reviewers recommend revisions. Reviewer #1 advises that for this to be an updated review, rather than a sequel, all 27 primary studies included in the previous 2017 analysis should be incorporated in this manuscript as well. In addition, both reviewers make several minor suggestions. Therefore, I invite you to revise and resubmit this manuscript.

Thank you - we found the reviewers’ comments and suggestions very helpful and have revised the manuscript accordingly. We have expanded the results section where we summarise the findings from the previous systematic review in order to provide a more comprehensive view of the main findings from that publication.

Reviewer #1: My main concern with this review relates to the fact that this is not an “updated review” (as the authors state) since only studies from 2017 onwards were included. Findings of the 2017 review (which was conducted by several of the same authors) are not included here – hence, this is not an update but merely a (minor) sequel. Therefore, all 27 primary studies included in the 2017 review (not just references 20 and 21 which were, at that time, unpublished) should be incorporated in this manuscript as well – so that this review reflects a comprehensive picture of *all* the evidence, not just that published after 2016. For example, the current manuscript omits data from many non-Asian countries (eg, Denmark, Finland, Germany, USA, Colombia, Jordan) that were identified in the 2017 review. This major point should be addressed in the revision.

We thank the reviewer for suggesting we provide a more detailed summary of the main findings from the previous review. We have amended the results section that summarised the review published by Gunnell et al to include the following information:

**“Summary of studies reported by Gunnell et al (10).**

In the previous systematic review, Gunnell et al (10) described 27 studies where HHP bans had taken place in 16 countries, which included data up to 2016 (8, 20, 21, 28-51). Five countries were LMIC (Sri Lanka, Bangladesh, Colombia, Jordan, and India) and 11 were HIC (Greece, South Korea, Taiwan, Denmark, UK, Finland, Germany, Hungary, Ireland, Japan, and USA).

Implementation of regulatory changes limiting access of HHPs led to a reduction in pesticide suicide in all countries with the exception of Greece, where no change in pesticide suicide rates were observed following the withdrawal of parathion and monocrotophos in Crete in 2003 (34). These included several European countries such as Denmark (39), Finland (42), Germany (43), Hungary (44, 45), Ireland (40, 46), and England (but not Scotland) (40, 41), as well as Japan (47) and the USA(49-51).

Overall suicide either decreased in three countries (i.e. Sri Lanka (8, 20, 31-33), Bangladesh (21), and Colombia (28)) or remained the same in one country (i.e. India (30)) following regulatory changes in all LMIC that reported on suicide rates. Results from HIC were mixed, with data from some countries showing an increase in overall suicides (i.e. Greece (34), Taiwan (37, 38), UK ) (40, 41), Ireland(40, 46), Finland (42), and USA (49-51)).

However, only five studies used appropriate time series analyses to take into account secular trends prior to the implementation of regulatory changes in access to HHPs (8, 20, 21, 28, 36). These studies were conducted in four LMIC counties (i.e. Sri Lanka, Bangladesh, Colombia) and in one HIC (i.e. South Korea). All but the study from Colombia (28) reported decreases in pesticide suicide and overall suicide rates following bans on HHPs.”

We have also revised the last paragraph of the introduction, to clarify the need for this review: “We build on previous literature by focusing on studies that used appropriate time series analyses to take into account secular trends prior to the interventions, since the majority of studies included in the previous systematic review on the same topic did not use appropriate methodology, limiting the strength of their conclusions (10).”

In the discussion, we have added two sentences to highlight the importance of this more current review, emphasizing that the focus on studies that used appropriate methodology (shown in Figures 3 and 4) strengthens the evidence on the effectiveness of bans on HHPs, since it takes into account secular trends.

“Importantly, findings from this systematic review not only provided an update on the findings from the previous review but also strengthened the quality of the evidence available, as the majority of the studies included in this systematic review used appropriate methodology to assess the effect of changes in regulations limiting access to HHPs on pesticide suicide rates.”

“The decrease [in rates] was particularly striking in Sri Lanka (50% for pesticide and 21% for overall suicides), Bangladesh (65% for pesticide and 25% for overall suicides), China (60% for pesticide and 45% for overall suicides), India (37% for pesticide and 10% for overall suicides), and Taiwan (44% for pesticide and 7% for overall suicides) (Figure 3-4, Supplementary materials), all of which used appropriate time series analyses methods. Findings from Gunnell et al (10) also showed decreases in pesticide and overall suicide rates in both HIC and LMIC; however, few of those studies used appropriate time series analyses (10), a considerable limitation of the previous review.”

Minor comment: the ‘findings’ section of the Abstract needs more detail. Please report effect sizes (with confidence intervals) from the primary studies and other relevant statistics, as well as risk of bias assessments of the included studies.

Thank you for this suggestion. We have added the following results derived from our analyses to the abstract:

“All nine studies in six Asian countries showed reductions in pesticide suicide rates following HHP bans (range 28.0% to 91.9%), of which six applied time series analyses to account for trends prior to the intervention (pesticide suicide rates ranged from 28.0% to 60.5%). Only five studies assessed overall suicides; of those, four reported decreases in overall suicide rates following the intervention, of which three used time series analysis (range 7% to 45.1%). Only one study had a low risk of bias in all domains, with five studies having high risk of bias in at least one of the domains.”

Estimates (with confidence intervals) derived from the primary studies are available in supplementary but we opted to include our results as opposed to those from the primary studies as they reflect the direct findings from our review, based on raw data provided by the authors of the primary studies included in the review.

Reviewer #2: Thank you for the opportunity to review this manuscript. It provides an important update and I am impressed that new data was obtained through correspondence. Please see some comments:

Thank you for your comment, we are glad that the reviewer found that our manuscript provides an important update on this topic.

Page 8 – “ordinal least squares” Is this meant to be ordinary least squares? Otherwise please briefly explain the analysis.

Thank you for highting this. It was in fact a typo and we have correctly accordingly.

Page 13 – “We mitigated against this be reference screening of all included...” Fix from be to by.

Thank you, we have corrected this.

Table 1 – In columns 3 and 4, some studies have a year and some don’t. I think including the year in all rows is useful, especially if the end of the study period represents the last year of available data following the interventions.

Thank you for this suggestion, we have included the year in the columns 3 and 4.

Figures – Use the same y axis values when comparing countries in Figures 3 and 4. Otherwise use some type of annotation to highlight when the values are different – eg when comparing China and Kerala in Figure 3, the relative slope decrease appears more dramatic in China but the absolute decrease in numbers would be smaller because of the way the axis is stretched out.

We agree that having different scales on the Y-axis is not ideal. However, should we have the same scale for all figures, it would be difficult to visualise some of the trends and slopes due to the fact that some countries have considerably higher rates than others. But we agree with the reviewer that this should be appropriately highlighted so that the reader is aware the scales are different for some of the figures and have added the following note to the footnote in Figures 3 and 4: “Please note that the scale for the Y-axis is different in each figure, as the age-standardised suicide rates vary considerably from country to country.”

Removal midline decimals if not standard journal style.

Thank you, we have corrected these.
